# NIV-Helmet in Severe Hypoxemic Acute Respiratory Failure

**DOI:** 10.1155/2015/456715

**Published:** 2015-04-27

**Authors:** Joana Martins, P. Nunes, C. Silvestre, C. Abadesso, H. Loureiro, H. Almeida

**Affiliations:** Pediatric Intensive Care Unit, Professor Doutor Fernando Fonseca Hospital, Lisbon, Portugal

## Abstract

Noninvasive ventilation (NIV) is a method to be applied in acute respiratory failure, given the possibility of avoiding tracheal intubation and conventional ventilation. A previous healthy 5-month-old boy developed low-grade intermittent fever, flu-like symptoms, and dry cough for 3 days. On admission, he showed severe respiratory distress with SpO_2_/FiO_2_ ratio of 94. Subsequent evaluation identified an RSV infection complicated with an increase of inflammatory parameters (reactive C protein 15 mg/dL). Within the first hour after NIV-helmet CPAP SpO_2_/FiO_2_ ratio increased to 157. This sustained improvement allowed the continuing of this strategy. After 102 h, he was disconnected from the helmet CPAP device. The NIV use in severe hypoxemic acute respiratory failure should be carefully monitored as the absence of clinical improvement has a predictive value in the need to resume to intubation and mechanical ventilation. We emphasize that SpO_2_/FiO_2_ ratio is a valuable monitoring instrument. Helmet interface use represents a more comfortable alternative for providing ventilatory support, particularly to small infants, which constitute a sensitive group within pediatric patients.

## 1. Background

Respiratory Syncytial Virus (RSV) is a major cause of viral respiratory tract infections in infants and children. The course of RSV infection is usually benign, with low mortality rates (estimated 2–5%), even for high-risk patients [1].

Acute respiratory failure (ARF) secondary to RSV infection has its prevalence estimated in 0.8–2.5%. However, this risk is difficult to access, since different groups within the pediatric population show different prevalence of ARF. For instance, 7.3–42% of children with bronchopulmonary dysplasia develop ARF in the context of RSV infection [2].

The mainstay of treatment for patients with severe ARF has been intubation and mechanical ventilation. However, noninvasive ventilation (NIV) can be considered as an alternative, given the possibility of avoiding direct complications of tracheal intubation and conventional ventilation [3, 4]. Nonetheless, it should be closely monitored in order to intubate and ventilate the patient in the presence of complications.

The main goals of using NIV, continuous positive airway pressure (CPAP) or bilevel positive airway pressure (BPAP), in patients with ARF are to improve oxygenation, to unload the respiratory muscles, and to relieve dyspnea, all of which should decrease the intubation rate.

Neuromuscular drive, inspiratory muscle effort, and relief of dyspnea significantly improve with BiPAP, compared to CPAP; however, oxygenation is better correlated with higher CPAP level (10 cm H_2_O) [4].

In this case report, we used the SpO_2_/FiO_2_ ratio as a descriptive measurement of ARF severity.

Oxygen saturation as measured by SpO_2_/FIO_2_ ratio has been demonstrated to correlate well with the PaO_2_/FiO_2_ ratio in both adult and pediatric studies, as long as SpO_2_ is between 80 and 97%. When SpO_2_ is over 97%, the oxyhemoglobin dissociation curve flattens and the SpO_2_/FiO_2_ ratio reliability is lost [5]. Rice et al. [6] validated this measurement for adults, by demonstrating that, for SpO_2_ ≤ 97%, PaO_2_/FiO_2_ ratio of 200 corresponds to SpO_2_/FiO_2_ ratio of 235.

## 2. Case Report

A previous healthy 5-month-old boy (10 Kg), with unknown familiar history, presents with low-grade intermittent fever, flu-like symptoms, and dry cough 3 days prior to admission. The symptoms progressively worsened, leading to an increased respiratory rate (RR: 80 bpm), respiratory distress (use of accessory respiratory muscles and subcostal and sternal retractions), and tachycardia (150–200 bpm). At pulmonary auscultation, subcrepitant rales were present with no increase of expiratory time.

On PICU admission, he presented SpO_2_ of 81% on room air (FiO_2_ 0.21) which responded poorly to oxygen administration, leading to the use of high oxygen flow mask (Venturi mask) with an initial FiO_2_ of 0.6 (SpO_2_ increases to the maximum of 91%), quickly ascending to FiO_2_ 1.0 (SpO_2_ of 92–94%: SpO_2_/FiO_2_ ratio 94).

Laboratory work-up revealed venous pH 7.430, pCO_2_ 34.8, HCO_3_ 22.6, and base excess of −1,3, microcytic hypocromic anemia, normal leucocyte count (leucocyte 6000/mm^3^, neutrophils 44.2%, and lymphocytes 41.7%), and a reactive protein C of 15 mg/dL. Respiratory secretions rapid immunological test identified RSV infection.

**Figure 1:**
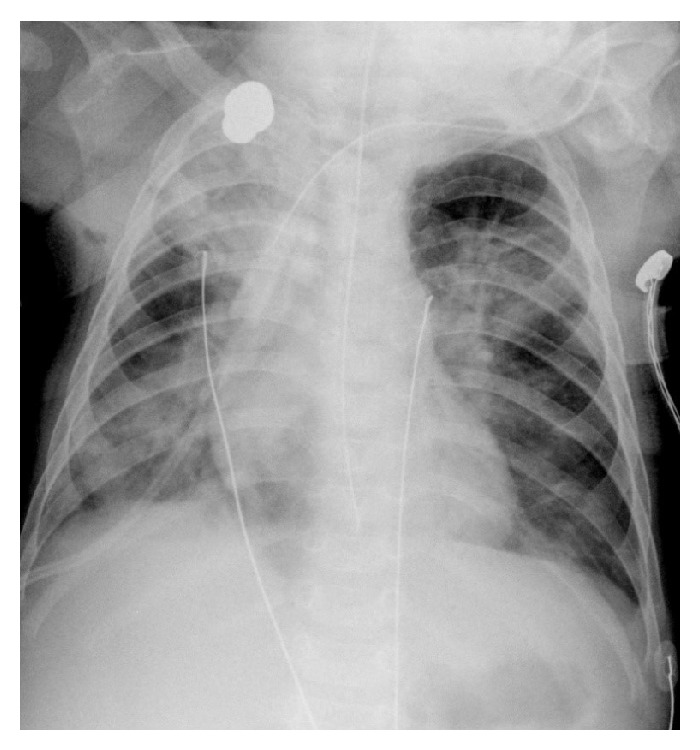
Thoracic X-ray on admission.

Thoracic X-ray (Figure 1) evaluation identified multiple hypotransparent foci, mainly at the upper right lobe, affecting three quadrants out of four, leading to the presumptive diagnosis of bronchopneumonia.

SpO_2_/FiO_2_ index was calculated, minimum of 94 (prior to NIV-CPAP implementation).

## 3. Treatment

**Figure 2:**
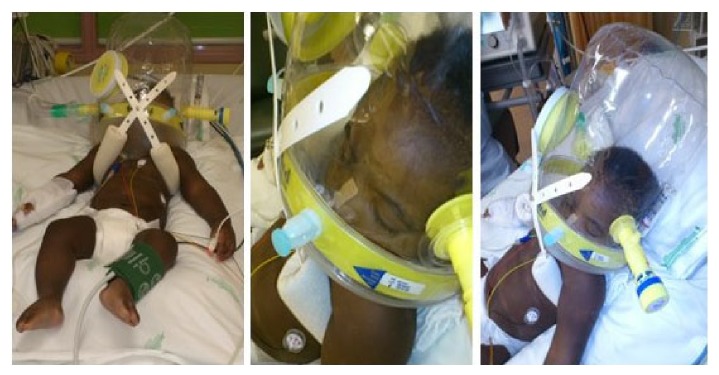
Helmet interface use with significant comfort for the patient.

The patient was then started on antibiotics, crystalline G penicillin 300 000 UI/Kg/day, and was connected to the helmet CPAP device (Figure 2), with an initial FiO_2_ 0.6 and PEEP 10 cm H_2_O.

Initially, there was a need for sedation boluses with midazolam (0.1 mg/kg q2 administration).

## 4. Follow-Up

**Figure 3:**
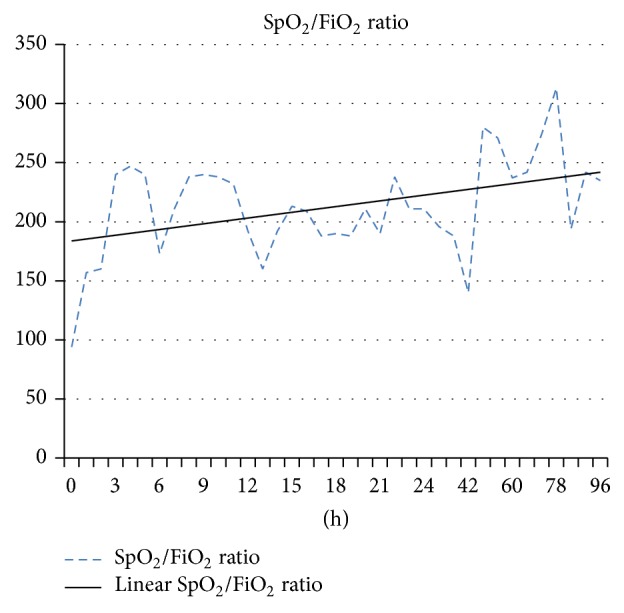
SpO_2_/FiO_2_ evolution after helmet CPAP connection.

Within the first hour after starting NIV, SpO_2_/FiO_2_ rose to 156, leading to SpO_2_/FiO_2_ of 240 by the second hour after connection (Figure 3).

By the same time, prominent improvement was also detected in respiratory rate and signs of respiratory effort (mainly intercostal and subcostal retractions).

In the first 48 h after admission, NIV suspension (need for secretions removal, for instance) caused a rapid oxygenation drop and a subsequent increase of the respiratory effort. Progressively, periods without ventilatory support became larger and more tolerated by the patients.

During the NIV administration period, there was no need for further sedative boluses. The patient was kept quiet using chloral hydrate 1-2 administrations a day, mainly in the first 48 hours of ventilatory support.

After 102 h of ventilatory support, he was definitely disconnected from the helmet CPAP device, keeping O_2_ administration through nasal prongs. He was transferred to a general pediatric ward at the 9th day after admission.

## 5. Discussion

NIV has shown positive effects in adult patients with different types of respiratory failure, being specially safe and effective for patients with hypercapnic ARF due to chronic obstructive pulmonary disease (COPD) exacerbation and hypoxemic ARF due to cardiogenic pulmonary oedema, community-acquired pneumonia, and immunocompromised patients with pulmonary infiltrates [7–9].

In pediatric population, NIV use in ARF patients showed a success rate between 57% and 92% [4], but this rate heterogeneity may be due to different age groups under analysis.

This patient represents a not so prevalent evolution for a RSV respiratory infection in a previously healthy child: within 3 days after the first symptoms, he progressed to hypoxemic ARF.

This patient was submitted to a numerous other tests (cultural analysis of blood and sputum) in order to identify other possible respiratory agents, but no other causes were determined. Nonetheless, the patient was started on antibiotics (intravenous penicillin 300 000 UI/Kg/day) right after admission, as bacterial coinfection was suspected mostly through the reactive protein C determination.

Much thought was dedicated to the possibility of this patient being an ARDS patient; however, according to the Berlin definition [10], he does not fulfill the diagnosis criteria, as the time between the beginning of the symptoms and the ARF was less than a week. Also we did not use PaO_2_ measurements to determine the PaO_2_/FiO_2_ ratio as recommended by this task force.

Essouri et al. [11] determined success rate in pediatric ARF patients under NIV of 73%. However, in the ARDS group, this success reached only 22%.

There are however other factors that can be used to predict NIV failure: lower age, apnea, bacterial coinfection [12], lower weight [13], higher clinical severity score (PRISM score, for instance), smaller respiratory and heart rate decrease within 1 hour after NIV, and increasing need for supplementary oxygen [13].

Mayordomo-Colunga et al. [13] suggested SpO_2_/FiO_2_ ratio of 193 one hour after NIV as the cut-off value under which endotracheal intubation should be considered in any ARF patient. In our case, 1 h after connection, our patient was still under the cut-off value, but the hemodynamic stability, the absence of gasometrical deterioration, a decrease in the FiO_2_ supplied, and a steady improvement in respiratory effort lead us to continue with NIV strategy under close surveillance.

By the second hour after NIV, the SpO_2_/FiO_2_ ratio was above the suggested cut-off value, granting us the support to maintain the chosen treatment. Nonetheless, severe ARF patients under NIV require close attention: Antonelli et al. refer to as much as seventy percent of the NIV failure of adult population being intubated within 48 hours [14]; however, Mayordomo-Colunga et al. showed that, in a pediatric population, the mean time of NIV support prior to failure and intubation was 13 to 16 h [11–13].

The choice between interfaces is mainly determined by the comfort that can be provided to the patient. In the adult population, face mask or helmet use have had the same rate of success (52% versus 49%) [14]. In pediatric patients, it is globally assumed that the different interfaces do not cause differences in the NIV success ratio, but it is only logical to assume that the difficulty in achieving a good ventilatory-child synchrony can be responsible for some of the failure cases. Most authors refer to the difficulty in fighting leakage, finding the appropriate mask size according to each child and preventing skin pressure lesions. Additionally, there is a comprehensible need of sedative use in these patients which can somehow compromise respiratory drive.

Skin pressure lesions are the NIV's more frequent complications: their incidence is estimated in 23% [15], and both facial and nasal interfaces share the same problems.

The helmet interface choice was mainly determined by this child's context: being a heavy and reactive 5 months old infant, we suspected that the adaptation to an oronasal interface would not be easy and would require sedative use.

The helmet adaptation was, nonetheless, simple and fast, with sedative use in boluses needed only in the first day. Two hours after NIV connection, the early response was notorious, with a significant reduction in FiO_2_ need and RR, but mainly the SpO_2_/FiO_2_ ratio increased in a steady way from 156–160 1 h after connection to above 200 <48 h after the connection.

## 6. Learning Points

- NIV use in severe ARF should be closely monitored.
- The first hours after NIV connection are crucial to determine the risk of NIV failure.
- Helmet use represents a more comfortable alternative for providing ventilatory support, particularly to small infants, which constitute a sensitive group within pediatric patients.
